# Supplementary material for: Insights into HIV-1 proviral transcription from integrative structure and dynamics of the Tat:AFF4:P-TEFb:TAR complex
Source: eLife. 2016 Oct 12;5:e15910. doi: 10.7554/eLife.15910 (PMC5072841; doi:10.7554/eLife.15910)
Supplement: Table 2—source data 1. — Twinning analysis by L-test in Ctruncate (A) and Xtriage indicate no twinning. Blue curve is acentric untwinned data, green curve acentric twinned data, and red curve observed data from Tat:AFF4:P-TEFb-TAR co-crystal. (B, C) Optimization of DEN refinement parameters using the web service for low resolution crystal structure refinement (https://portal.sbgrid.org/d/apps/den/). (D) Log-file of twinning analysis in CNS. DOI: http://dx.doi.org/10.7554/eLife.15910.011 [file elife-15910-table2-data1.pdf]

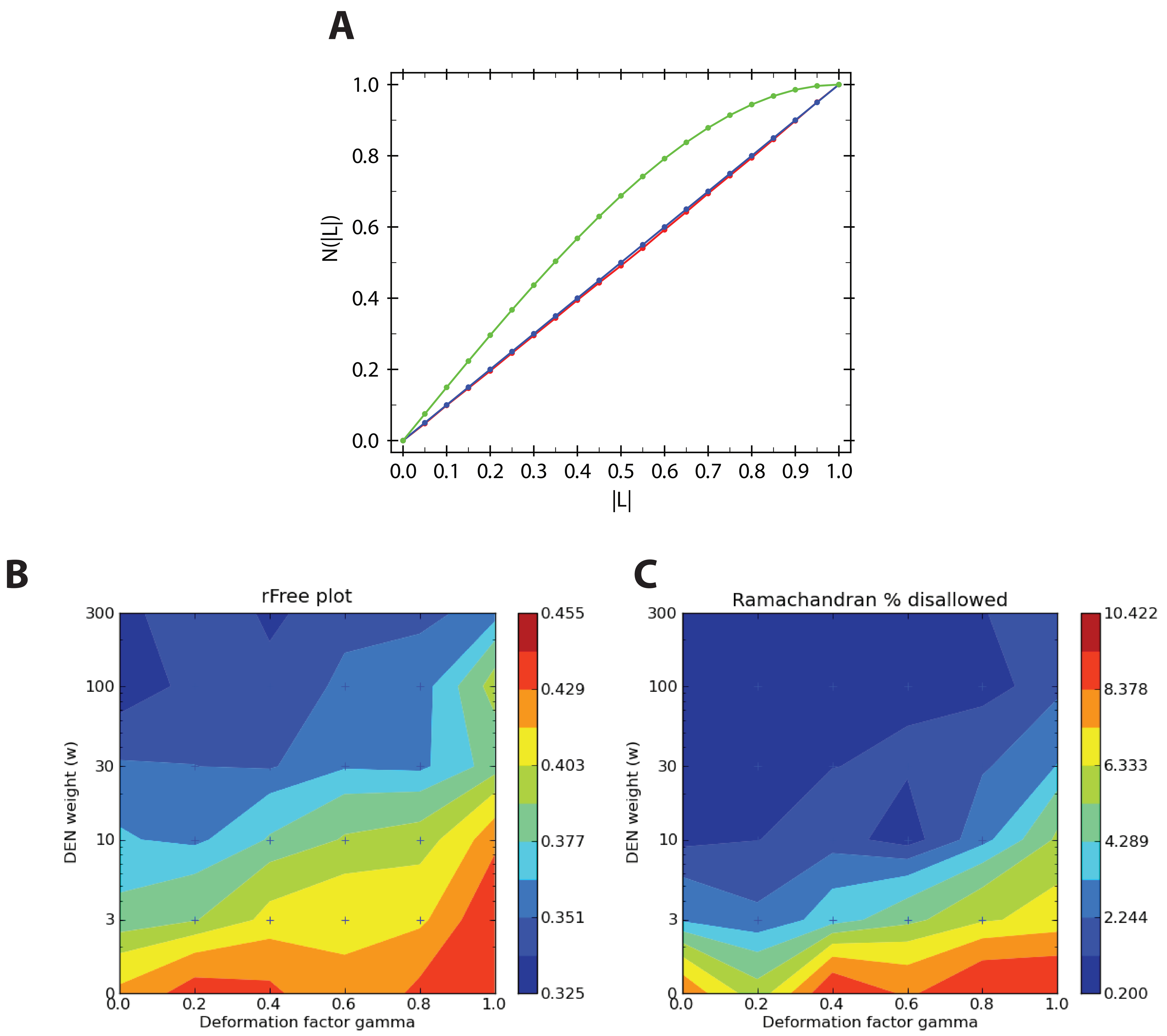


**D**

```
Log-file of twinning analysis in CNS


>>>> results of twinning detection
>>>> resolution: 500.0 - 5.9 A
>>>> sg= P3(2)21 a= 146.87 b= 146.87 c= 103.75 alpha= 90 beta= 90 gamma= 120
>>>> reflection file 1 : scaled_new.cns
>>>> reflections with |Fobs|/sigma_F < 0.0 rejected
>>>> reflections with |Fobs| > 10000 * rms(Fobs) rejected
>>>> theoretical total number of refl. in resol. range:     3596 ( 100.0 % )
>>>> number of unobserved reflections (no entry):              1 (   0.0 % )
>>>> number of reflections rejected:                           0 (   0.0 % )
>>>> total number of reflections used:                      3595 ( 100.0 % )
>>>> number of reflections in working set:                  3595 ( 100.0 % )

===============================================================================

              testing for perfect twinning

 column  1:   bin number
 columns 2:   upper resolution limit
 columns 3:   lower resolution limit
 column  4:   number of reflections in bin
 column  5:   average resolution in bin
 column  6:    <|I|^2>/(<|I|>)^2
 column  7:   (<|F|>)^2/<|F|^2>
 column  8:   fraction of theoretically complete data

  <|I|^2>/(<|I|>)^2 is 2.0 for untwinned data, 1.5 for twinned data
 (<|F|>)^2/<|F|^2> is 0.785 for untwinned data, 0.865 for twinned data

 #bin | resolution range | #refl |
   1 11.29  500.01     390    16.2548   6.9180   0.6679   0.7117
   2  8.96   11.29     431     9.9532   3.1956   0.6935   0.8225
   3  7.83    8.96     423     8.3476   4.0320   0.6877   0.8426
   4  7.11    7.83     443     7.4507   4.5658   0.6947   0.8619
   5  6.60    7.11     444     6.8389   2.3690   0.8300   0.8792
   6  6.21    6.60     434     6.3962   1.9642   0.8926   0.8697
   7  5.90    6.21     448     6.0480   1.7855   0.8997   0.8889

--------------------------averages-over-all-bins----------------------------
   <|I|^2>/(<|I|>)^2 =    3.4875 (2.0   for untwinned, 1.5   for twinned)
  (<|F|>)^2/<|F|^2>  =    0.7690 (0.785 for untwinned, 0.865 for twinned)
----------------------------------------------------------------------------

===============================================================================

    testing for partial twinning (using statistical method of Yeates)


>>>> testing for twinning operator= -h,-k,l


    <H> =  0.50707: twinning fraction= -.007 (434 reflections used)
   <H^2> =  0.34743: twinning fraction= -.010 (434 reflections used)

===============================================================================
```

**Table 2 Source data 1**. Diffraction data and refinement analysis. Twinning analysis by L-test in Ctruncate (**A**) and Xtriage indicate no twinning. Blue curve is acentric untwinned data, green curve acentric twinned data, and red curve observed data from Tat:AFF4:P-TEFb-TAR co-crystal. (**B, C**) Optimization of DEN refinement parameters using the web service for low resolution crystal structure refinement (https://portal.sbgrid.org/d/apps/den/).
(**D**) Logfile of twinning analysis in CNS.
